# Transposable element dynamics in *Xenopus laevis* embryogenesis: a tale of two coexisting subgenomes

**DOI:** 10.1186/s13100-025-00350-3

**Published:** 2025-04-09

**Authors:** Edith Tittarelli, Elisa Carotti, Federica Carducci, Marco Barucca, Adriana Canapa, Maria Assunta Biscotti

**Affiliations:** 1 Dipartimento di Scienze della Vita e dell’Ambiente, Università Politecnica delle Marche, Via Brecce Bianche, Ancona, 60131 Italy; 2 Scuola Universitaria Superiore Pavia – IUSS, Piazza della Vittoria n.15, Pavia, 27100 Italy

## Abstract

The African clawed frog *Xenopus laevis* has an allotetraploid genome consisting of two subgenomes referred as L relating to the Long chromosomes and S relating to the Short chromosomes. While the L subgenome presents conserved synteny with *X. tropicalis* chromosomes, the S subgenome has undergone rearrangements and deletions leading to differences in gene and transposable element (TE) content between the two subgenomes. The asymmetry in the evolution of the two subgenomes is also detectable in gene expression levels and TE mobility. TEs, also known as “jumping genes”, are mobile genetic elements having a key role in genome evolution and gene regulation. However, due to their potential deleterious effects, TEs are controlled by host defense mechanisms such as the nucleosome remodeling and deacetylase (NuRD) complex and the Argonaute proteins that mainly modify the heterochromatin environment. In embryogenesis, TEs can escape the silencing mechanisms during the maternal-to-zygotic transition when a transcriptionally permissive environment is created. Moreover, further evidence highlighted that the reactivation of TEs during early developmental stages is not the result of this genome-wide reorganization of chromatin but it is class and stage-specific, suggesting a precise regulation. In line with these premises, we explored the impact of TE transcriptional contribution in six developmental stages of *X. laevis*. Overall, the expression pattern referred to the entire set of transcribed TEs was constant across the six developmental stages and in line with their abundance in the genome. However, focusing on subgenome-specific TEs, our analyses revealed a distinctive transcriptional pattern dominated by LTR retroelements in the L subgenome and LINE retroelements in the S subgenome attributable to young copies. Interestingly, genes encoding proteins involved in maintaining the repressive chromatin environment were active in both subgenomes highlighting that TE controlling systems were active in *X. laevis* embryogenesis and evolved symmetrically.

**Supplementary Information:**

The online version contains supplementary material available at 10.1186/s13100-025-00350-3.

## Introduction

The African clawed frog *Xenopus laevis* is an embryological model organism that has contributed extensively to the understanding of vertebrate development. Its allotetraploid genome evolved from a hybrid originated from two closely related diploid *Xenopus* species about 17–18 Mya [1]. Therefore, two subgenomes can be distinguished and referred as L related to Long chromosomes and as S related to Short chromosomes. Although the two subgenomes share the same nucleus, they evolved asymmetrically as separated compartments [2]. Indeed, the L subgenome shows a conserved synteny with *X. tropicalis* chromosomes, while the S subgenome experienced intra-chromosomal rearrangements and substantial deletions leading to differences in gene and transposable element (TE) content between the two subgenomes [1]. Moreover, changes are found also in gene expression levels and TE mobility in relation to a distinct impact of transcriptional silencing mechanisms that influence the heterochromatin environment [3–5]. TEs are genetic elements able to move throughout the genome using transposition mechanisms that are based on a DNA intermediate molecule in the case of DNA transposons and an RNA molecule in the case of retroelements. These latter can be further distinguished in Long Terminal Repeats (LTR) and non-LTR retrotransposons (including Short Interspersed Nuclear Elements (SINEs) and Long Interspersed Nuclear Elements (LINEs)) whether or not they have direct LTR-flanking sequences. Although the effects of TE mobility are mainly neutral, it is recognized that they can generate chromosome rearrangements, new genes and regulatory elements contributing positively into genome plasticity. However, as consequence of transposition, TEs can cause negative effects that are counteracted by genomes through the involvement of silencing mechanisms. These are mainly based on the deposition of repressive epigenetic marks from heterochromatin proteins that determine an increase in the heterochromatinization level of TE-containing sequences. Among TE host defense mechanisms, proteins of the Argonaute family (AGO and PIWI) use small RNAs (as siRNA and piRNA) to target TE transcripts to degrade them, to repress their translation, and to form heterochromatin [6]. The nucleosome remodeling and deacetylase (NuRD) complex contains proteins as the histone deacetylases that deposit repressive epigenetic marks at histone tails [3].

Despite their potential negative impact, TEs are not completely silenced during early development [7–10]. Indeed, an increasing number of papers has reported that TEs play an essential role for normal development [11] and specific retroelements can participate in rewiring regulatory networks and driving evolutionary innovations [5, 12]. In *X. laevis*, multiple TE silencing mechanisms coexist and their transcription pattern has been reported in gonadal and somatic tissues [13].

Since each *X. laevis* subgenome has distinctive molecular genetic characteristics, we explored the TE landscape at genome and subgenome level as well as the transcriptional contribution and activity of these mobile elements in six developmental stages (zygote, blastula, gastrula, neurula, tailbud, and early tailbud) analyzing available RNA-Seq data [14]. Considering subgenome-specific TEs, our results evidenced a higher transcriptional contribution of those located in the L subgenome than those present in the S subgenome. Moreover, a distinctive pattern was observed between the two subgenomes, characterized by a dominance of LTR retroelements in the L subgenome and LINE retroelements in the S subgenome across most of the analysed developmental stages. The Kimura landscapes showed that the expressed specific TEs of both subgenomes were mainly young copies. Finally, the analyses on the transcriptional levels of genes encoding proteins involved in maintaining the repressive chromatin environment suggested an equal contribution of both *X. laevis* subgenomes in controlling TE activity during embryogenesis.

## Materials and methods

### Genome and subgenome transposable element landscapes of *X. laevis*

*X. laevis* genome was downloaded from the public database NCBI GenBank (https://www.ncbi.nlm.nih.gov/genome/) under the accession number GCA_017654675.1. The species-specific *de novo* TE library was build following the pipeline described in Carotti et al. 2021 [15] (see SupplementarFig. . 1). Briefly, RepeatScout v 1.0.6 [16] was used to identify TEs and the generated “build_lmer_table” was used by RepeatMasker v 4.1.0 (http://www.repeatmasker.org/cgi-bin/WEBRepeatMasker, accessed on 5 March 2024). Filtering steps were applied to remove sequences not identified as TEs: repeats accounted for less than 10 times were removed and, using a threshold e-value of 1 × 10^− 50^, BLASTX [17] search against the Uniprot–Swissprot database [18] and Interproscan v5.34–73.0 [19] were performed. The discarded elements were searched by HMMER to identify integrase, reverse transcriptase, and transposase domains with e-value lower than 1 × 10^− 5^ and elements containing these domains were included in the TE library. Finally, the remaining sequences were classified using TEclass- 2.13 (https://www.bioinformatics.uni-muenster.de/tools/teclass/index.hbi?, accessed on 11 March 2024). The obtained library was employed by RepeatMasker to mask *X. laevis* genome and each subgenome. To identify subgenome-specific TEs, we retrieved and removed the TE sequences shared between the two output files of RepeatMasker obtained for each subgenome (SupplementarFig. . 1). TE age and transposition history were estimated at genome and subgenome level. The rate of transitions and transversions was calculated between genome/subgenome and TE consensus obtained from the library applying the scripts “calcDivergenceFromAlign.pl” and “createRepeatLandscape.pl” included in the RepeatMasker package.

### Transcriptome reconstruction and completeness evaluation of six developmental stages in *X. laevis*

*X. laevis* RNA-seq raw data of six different developmental stages (zygote, blastula, gastrula, neurula, tailbud, and early tailbud) were obtained from the Sequence Read Archive (SRA) (https://www.ncbi.nlm.nih.gov/sra, accessed on 3 April 2024) under the accession numbers SRP431184 reported in supplementary Table 1. For each stage, raw paired-end reads were imported into the CLC Genomics Workbench v.12 (Qiagen, Hilden, Germany), trimmed to remove sequencing adapters, the low-quality bases/reads, and then assembled *de novo* using the default parameters. The completeness of the *de novo* assembled transcriptomes was evaluated using BUSCO v.5, with the Tetrapoda OrthoDB v.10 database as a reference [20].

### TE transcriptional contribution, Kimura distance-based TE age distribution, and differentially expressed TEs

To evaluate the TE transcriptional contribution, we first identified TEs in the *de novo* assembled transcriptomes with RepeatMasker v.4.1.0 (http://www.repeatmasker.org/cgi-bin/WEBRepeatMasker, accessed on 10 April 2024) using a *X. laevis* custom TE genome library described above. For these sequences, values derived from mapping procedure of triplicate trimmed reads against the transcriptomes were used to calculated their total transcriptional contribution as percentage for each developmental stage. However, this approach did not allow distinguishing mapping values deriving from reads obtained from TE copies present in both subgenomes. Therefore, subgenome-specific TE libraries were used to mask each transcriptome and mapping values related to subgenome-specific TEs were retrieved from the performed mapping. The transcriptional activity of these elements was calculated as TPM following the methodology described for genes of interest. Moreover, only for transcribed TE elements, the RepeatMasker package was used to obtain Kimura distances, which reflect the rates of transition and transversions.

We used TEtranscripts v2.2.3 [21] to estimate differentially expression TEs (DETEs) between pairwise comparisons of considered developmental stages, as follow: Blastula vs. Zygote, Gastrula vs. Blastula, Neurula vs. Gastrula, Tailbud vs. Gastrula, and Early tailbud vs. Tailbud. To perform this analysis the input files were: the BAM file of triplicates sorted by position with SAMtools [22]; the gene annotation file downloaded from NCBI (GCF_017654675.1); the TE annotation file generated from RepeatMasker output file. DETEs having Log2 Fold Change >|2| and the statistically significant threshold -Log10 (p-adj) = 0.05 were graphically represented with Rstudio [23] using ggplot2 [24] and dplyr [25] packages. Gene expression tables (Supplementary Table 2), gtf file (Supplementary File 1) and TE consensus library (Supplementary File 2) were reported as supplementary materials.

### Transcriptional activity of genes encoding proteins involved in TE controlling systems

Genes of interest were searched in CDS file downloaded from https://www.xenbase.org/xenbase/static-xenbase/ftpDatafiles.jsp. The gene set includes: for heterochromatinization: *chromobox homolog 1* (*cbx1*), *chromobox homolog 3* (*cbx3*), *chromobox homolog 5* (*cbx5*), *DNA (cytosine-5-)-methyltransferase 1* (*dnmt1*), *DNA (cytosine-5-)-methyltransferase 3 alpha* (*dnmt3α*) and, *SET domain bifurcated histone lysine methyltransferase 1* (*setdb1*); for NuRD complex *chromodomain helicase DNA binding protein 3* (*chd3*), *chromodomain helicase DNA binding protein 4* (*chd4*), *histone deacetylase 1* (*hdac1*), *histone deacetylase 2* (*hdac2*), *methyl-CpG binding domain protein 2* (*mbd2*), *methyl-CpG binding domain protein 3* (*mbd3*), *metastasis associated 1* (*mta1*), *metastasis associated 1 family*,* member 2* (*mta2*), *GATA zinc finger domain containing 2a* (*gatad2a*), *GATA zinc finger domain containing 2b* (*gatad2b*), *retinoblastoma binding protein 4* (*rbbp4*), *retinoblastoma binding protein 7* (*rbbp7*) and, *Tripartite Motif Containing 28* (*trim28*); for *Argonaute* gene subfamily: *Argonaute RISC Component 1* (*ago1*), *Argonaute RISC Component 2* (*ago2*), *Argonaute RISC Component 3* (*ago3*), *Argonaute RISC Component 4* (*ago4*). In addition, among all ZNF genes, those containing the KRAB domain were searched. For all considered genes, the transcriptional activity values reported as Transcript per Million (TPM) were calculated using the pipeline described in our previous work [26].

## Results

### *X. laevis* mobilome and transcriptional contribution in early development

**Fig. 1:**
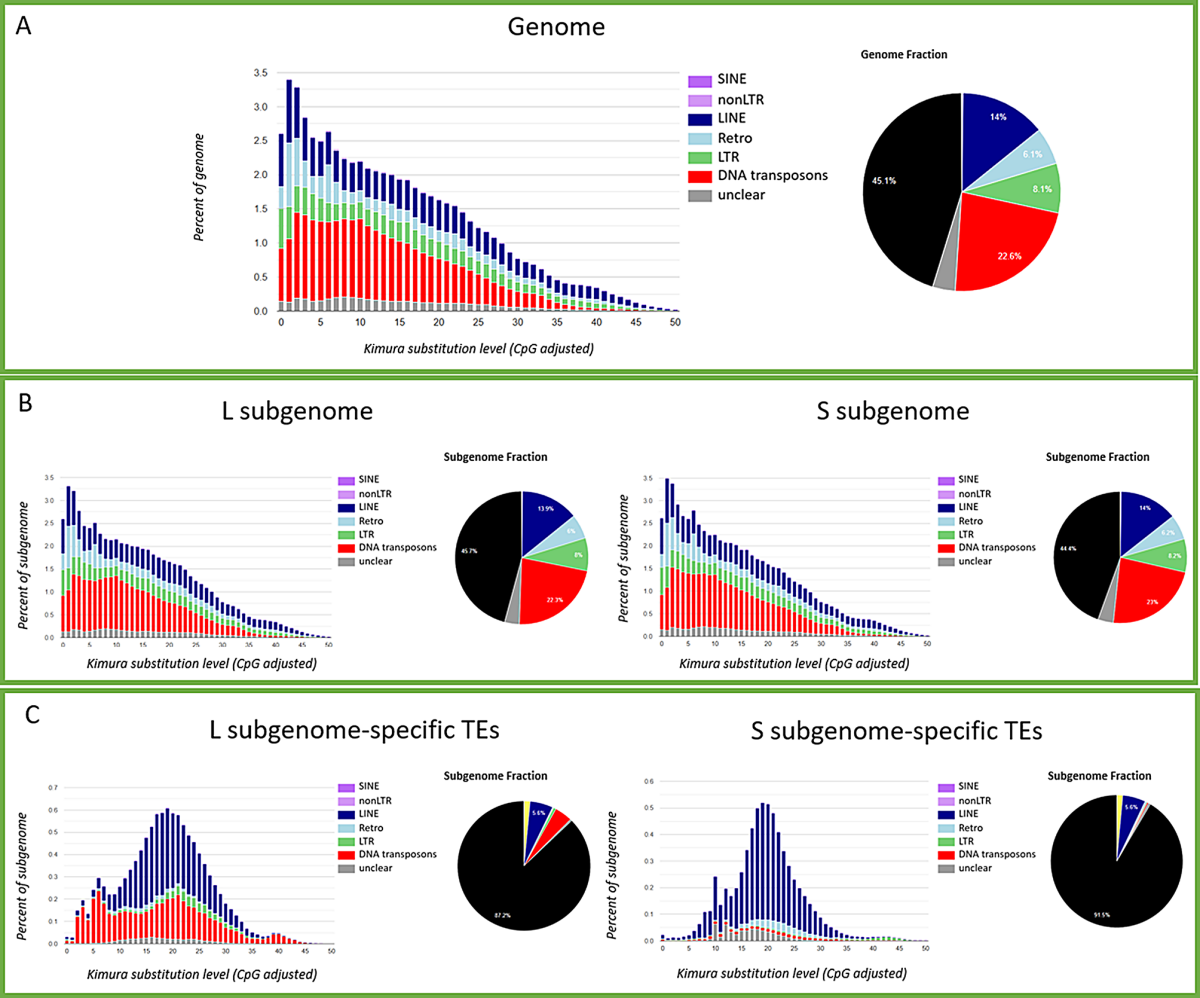
Repeat contribution and Kimura landscapes in the *Xenopus laevis* genome and subgenomes. **A**. On the left, the repeat landscape plot obtained by Kimura distance-based copy divergence analyses of TEs in *X. laevis* total genome. On the right, aerogram with TE type percentage of genome composition. **B**. Aerograms with TE type percentage of each subgenome compositions and repeat landscape plots obtained by Kimura distance-based copy divergence analyses of TEs in the *X. laevis* L and S subgenomes, respectively. **C**. Aerograms with subgenome-specific TE type percentage and repeat landscape plots obtained by Kimura distance-based copy divergence analyses of subgenome-specific TEs, respectively. In all panels, “unclear” means TEs that are not specifically classified as DNA transposons, LINE, SINE, and LTR retrotransposons, “non-LTR” retrotransposons are referred to retrotransposons that are not specifically classified as LINE or SINE retrotransposons, and “Retro” is referred to retrotransposons that are not specifically classified as LINE, SINE, LTR or non-LTR retrotransposons

The RepeatMasker analysis of *X. laevis* genome highlighted about 55% of TE content (Fig. 1A). DNA transposons represented the dominant TE type followed by LINE and LTR retrotransposons while SINE retrotransposons were very limited. The Kimura distance analysis showed the presence of one amplification burst between 0 and 5 K-values and the largest part of TE copies was below a K-value of 25 (Fig. 1A). The RepeatMasker analyses performed in each subgenome showed that about 55% was made up of TEs and the composition in terms of TE type reflected that observed at total genome level (Fig. 1B). The landscapes of Kimura distance analyses performed on the L and S subgenomes were similar to that obtained for total genomic TE copies. In addition, analyzing the subgenome-specific TEs, we identified 368 and 109 TEs in the L and S subgenomes, respectively. Among the L subgenome-specific TEs, the most abundant were LINE retrotransposons and then LTR retrotransposons and DNA transposons. LINE retrotransposons prevailed also among the S subgenome-specific TEs, followed by DNA transposons and LTR retrotransposons. At family level *Harbinger*, *hAT*, *Kolobok*, *Mariner*, *MuDR*, *Politon*, *CR1*, *L1*, *Copia*, *DIRS*, *Gypsy*, and *Penelope* were represented in the specific TEs of both subgenomes; *Helitron* and *Zisupton* were identified only in the S subgenome-specific TEs; *Cripton*, *PiggyBAC*, *Transib*, *Jockey*, *RTE*, and *Bel* were retrieved in the L subgenome-specific TEs. The Kimura landscape of the L subgenome-specific TEs was characterized by three amplifications bursts under a K-value of 25, while for S subgenome-specific TEs only two picks were observed (Fig. 1C).

**Fig. 2:**
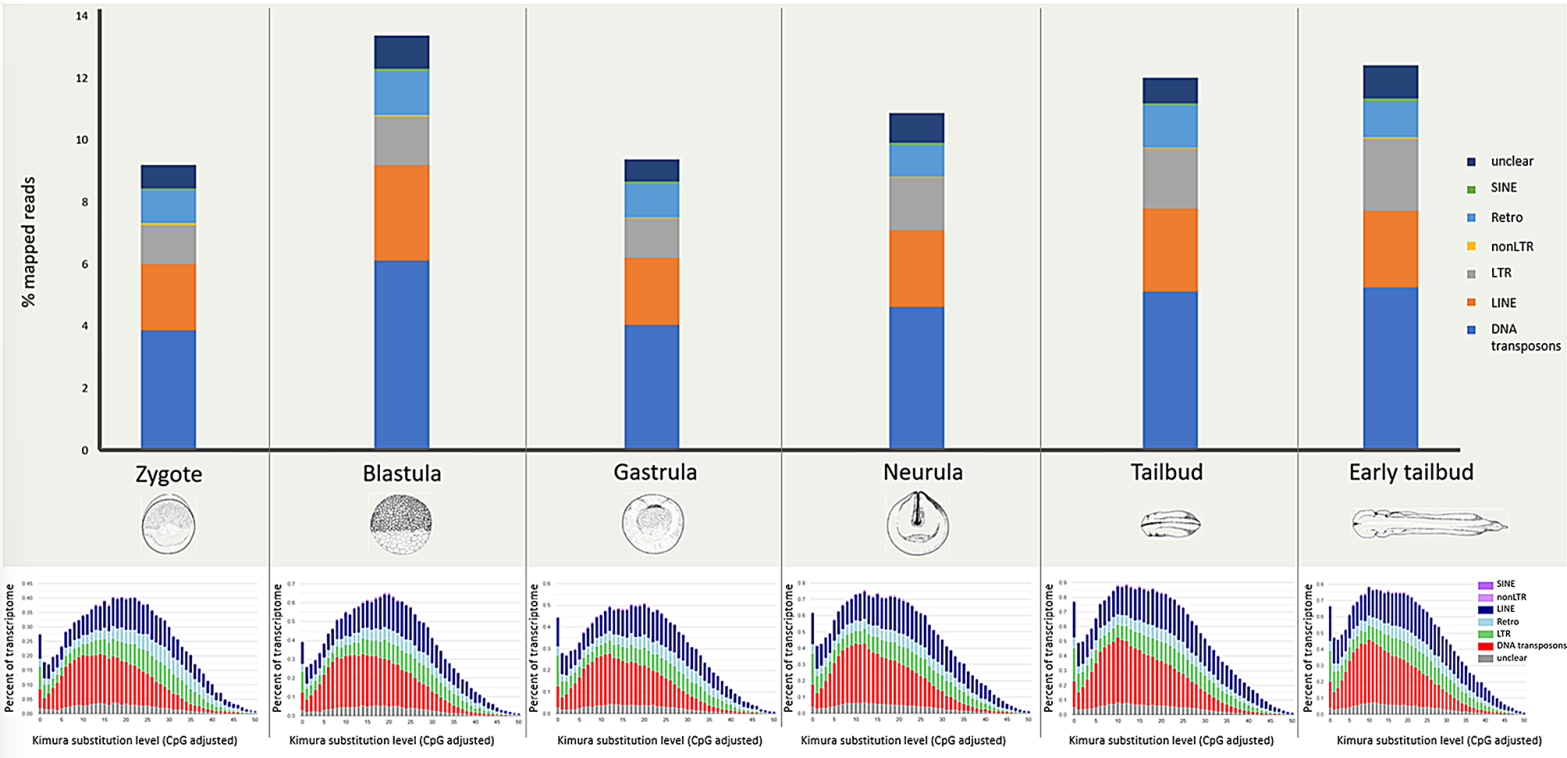
Transcriptional contribution of TEs in *X. laevis* early development and Kimura landscapes. In the upper side, the transcriptional contribution of TEs as percentage of mapped reads was reported in six developmental stages. In the lower side, the repeat landscape plots obtained by Kimura distance-based copy divergence analyses of transcribed TEs were reported. “Unclear” means TEs that are not specifically classified as DNA transposons, LINE, SINE, and LTR retrotransposons, “non-LTR” retrotransposons are referred to retrotransposons that are not specifically classified as LINE or SINE retrotransposons, and “Retro” is referred to retrotransposons that are not specifically classified as LINE, SINE, LTR or non-LTR retrotransposons

The total transcriptional contribution of TEs was evaluated in six stages of *X. laevis* early development: zygote, blastula, gastrula, neurula, tailbud, and early tailbud (Fig. 2). In terms of percentage of TE mapped reads, the highest value was recorded in blastula stage. Indeed, the transcriptional profile was characterized by an increase from zygote to blastula, followed by a decrease in gastrula and then a progressive intensification of TE expression up to early tailbud. Across all analysed stages, the major impact was due to DNA transposons followed by LINE and LTR retrotransposons, while the transcriptional contribution of SINE retrotransposons was very limited. The Kimura landscapes of expressed TEs were similar in all *X. laevis* developmental stages considered (Fig. 2).

**Fig. 3:**
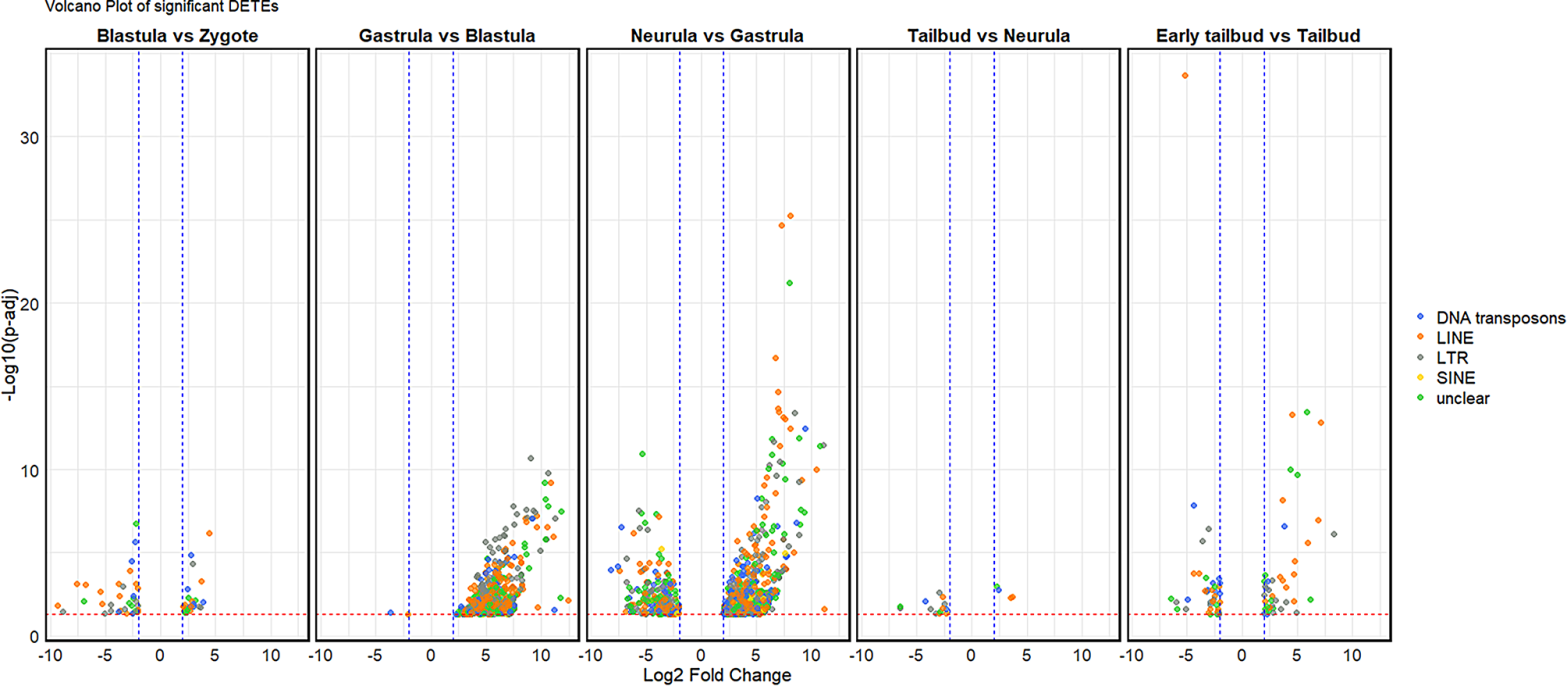
Volcano plot of differentially expressed TEs (DETEs) during developmental stages of *X. laevis*. From left to right, each graph reports the results of comparisons between the considered developmental stages (Blastula vs. Zygote, Gastrula vs. Blastula, Neurula vs. Gastrula, Tailbud vs. Gastrula, and Early tailbud vs. Tailbud). The blue dashed lines indicate the significant thresholds for Log2 Fold Change >|2|, while the red dashed the statistically significant threshold (-Log10 (p-adj) = 0.05). Each dot represents a single DETE and the color indicates the TE typology (DNA transposons in blue, LINE retroelements in orange, LTR retroelements in grey, SINE retroelements in yellow, Unknown, referred to TEs that are not classified as the previous typologies, in green)

Findings obtained performing TEtranscripts highlighted that TEs showing differential expression in the five comparisons carried out belong to DNA transposons, LINE and LTR retrotransposons and, unclear. The number of DETEs was limited in the comparisons Blastula vs. Zygote, Tailbud vs. Neurula, and Early tailbud vs. Tailbud while a higher number of DETEs was recorded in Gastrula vs. Blastula and Neurula vs. Gastrula. In particular in the former comparison the majority of DETEs showed an upregulation in Gastrula and in the latter comparison the number of up and down regulated DETEs was comparable (Fig. 3, Supplementary Fig. 2).

**Fig. 4:**
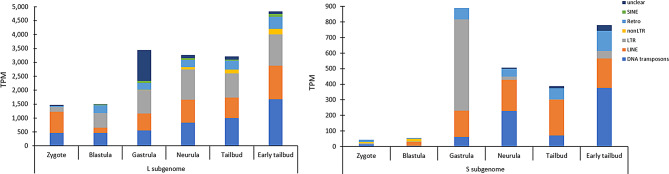
Cumulative transcriptional activity of subgenome-specific TEs. On the left, cumulative transcriptional levels of L subgenome-specific TEs were reported; On the right, cumulative transcriptional levels of S subgenome-specific TEs were reported. “Unclear” means TEs that are not specifically classified as DNA transposons, LINE, SINE, and LTR retrotransposons, “non-LTR” retrotransposons are referred to retrotransposons that are not specifically classified as LINE or SINE retrotransposons, and “Retro” is referred to retrotransposons that are not specifically classified as LINE, SINE, LTR or non-LTR retrotransposons

Considering subgenome-specific TEs, our results evidenced a higher activity of TEs specific of L subgenome than those specific of S subgenome. Moreover, although the TE type composition of these two sets of subgenome-specific TEs was similar (Fig. 1B), a distinctive pattern was observed across the analysed developmental stages. In general, regarding L subgenome-specific TEs, LTR retrotransposons were expressed from blastula to early tailbud; LINE retrotransposons were expressed in all stages with a major contribution in zygote and early tailbud; DNA transposons showed expression in all stages with a lower contribution in the first three developmental stages. Regarding S subgenome-specific TEs, LTR retrotransposons were considerably expressed in gastrula, LINE retrotransposons from blastula to early tailbud with a consistent impact in the tailbud stage, and DNA transposon expression was remarkable in neurula and early tailbud stages (Fig. 4, Supplementary Fig. 3).

**Fig. 5:**
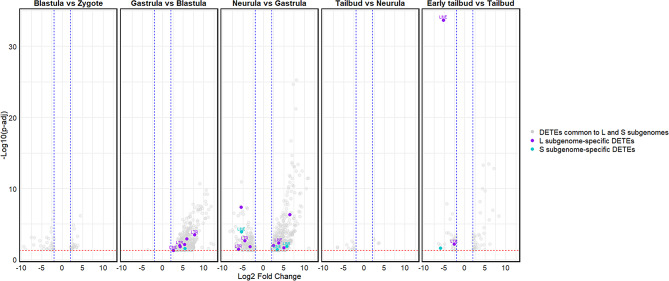
Volcano plot of subgenome-specific differentially expressed TEs (DETEs) during developmental stages of *X. laevis*. From left to right, each graph reports the results of comparisons between the considered developmental stages (Blastula vs. Zygote, Gastrula vs. Blastula, Neurula vs. Gastrula, Tailbud vs. Gastrula, and Early tailbud vs. Tailbud). The blue dashed lines indicate the significant thresholds for Log2 Fold Change >|2|, while the red dashed the statistically significant threshold (-Log10 (p-adj) = 0.05). DETEs common to L and S subgenomes are reported in light grey; L subgenomes-specific DETEs are colored in purple; S subgenome-specific DETEs are colored in turquoise. The colored dots without label are referred to “unclear”, TEs that are not specifically classified as DNA transposons, LINE, SINE, and LTR retrotransposons

The comparisons performed using TEtranscripts showed a limited number of subgenome-specific DETEs and these elements were mainly attributable to LINE and LTR retrotransposons (Fig. 5).

**Fig. 6:**
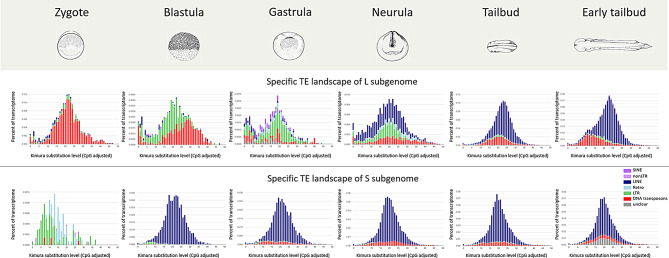
Kimura landscapes of transcribed subgenome-specific TEs during *X. laevis* development. Repeat landscape plots obtained by Kimura distance-based copy divergence analyses of L and S subgenome-specific TEs transcribed in six developmental stages. “Unclear” means TEs that are not specifically classified as DNA transposons, LINE, SINE, and LTR retrotransposons, “non-LTR” retrotransposons are referred to retrotransposons that are not specifically classified as LINE or SINE retrotransposons, and “Retro” is referred to retrotransposons that are not specifically classified as LINE, SINE, LTR or non-LTR retrotransposons

In zygote stage, the L subgenome-specific TEs expressed were mainly young copies of DNA transposons. This contribution decreased in blastula in which the transcriptional contribution was also due to recent copies of LTR retrotransposons and few copies of LINE retrotransposons. In gastrula, the impact of DNA transposons was further reduced and the contribution of LTR and LINE retrotransposons remained appreciable as that of SINE retrotransposons. In neurula, young copies of DNA transposons, LINE and LTR retrotransposons were expressed. However, these latter were reduced in the last two stages. The Kimura landscapes of the transcribed S subgenome-specific TEs showed young copies of LTR retrotransposons in zygote stage and mainly LINE retrotransposons from blastula to early tailbud (Fig. 6).

### Transcriptional activity of TE controlling systems in *X. laevis* early development

**Fig. 7:**
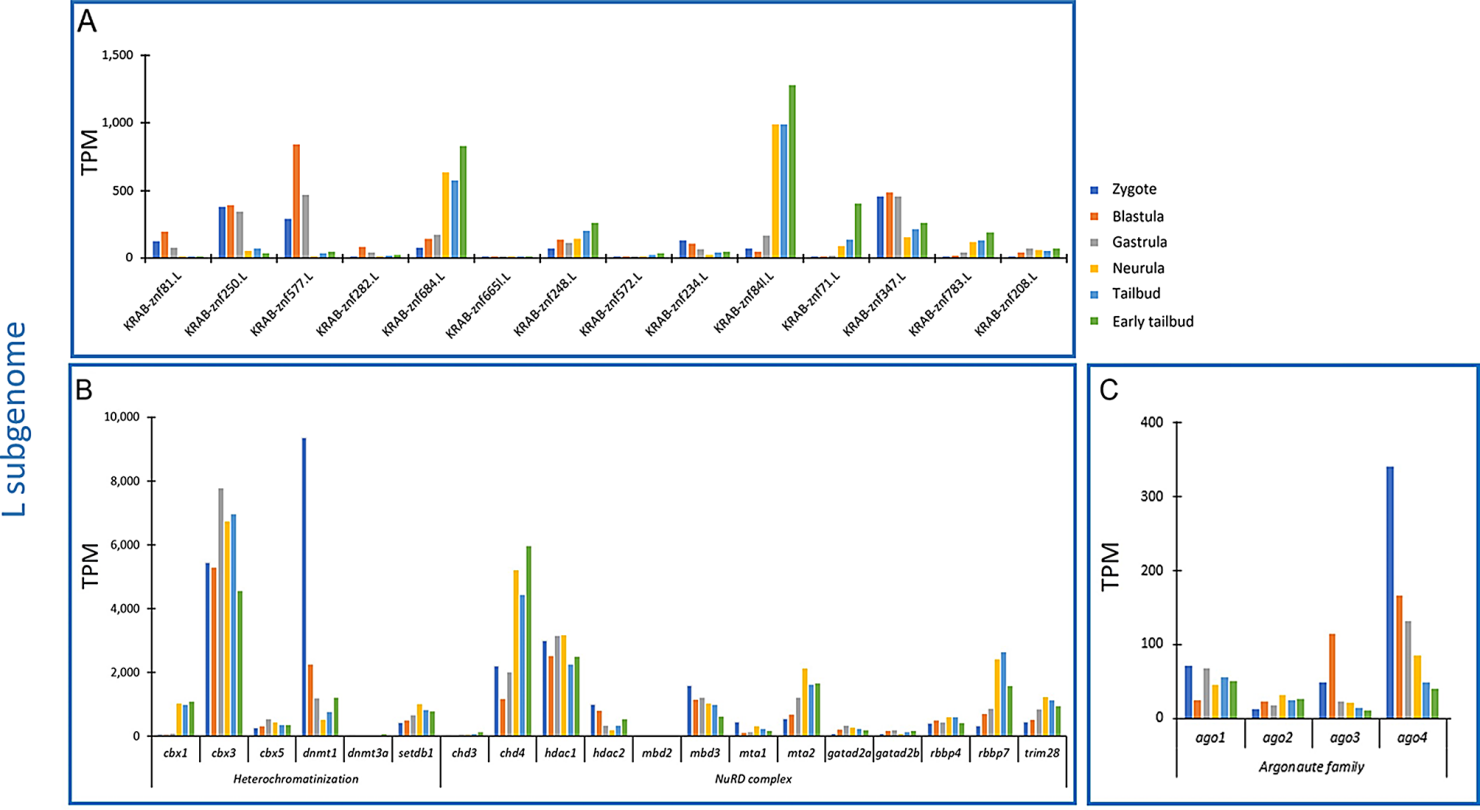
Transcriptional activity of genes involved in TE silencing mechanisms during *X. laevis* early development in the L subgenome. **A**. Expression values of genes encoding KRAB-ZFPs. **B**. Expression values of genes encoding proteins involved in the formation of the heterochromatin and NuRD complex. **C**. Expression values of genes encoding proteins of Argonaute subfamily

**Fig. 8:**
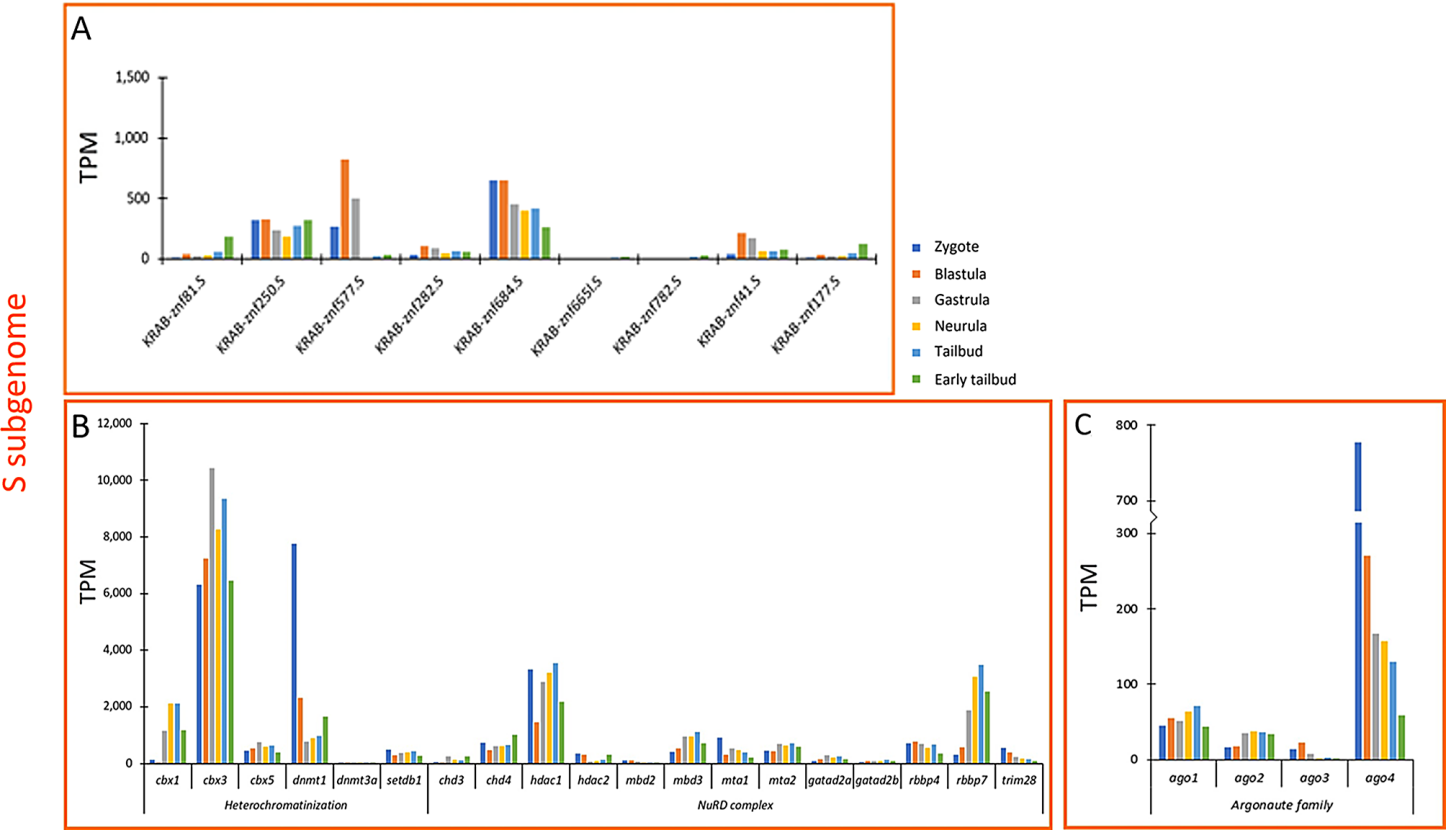
Transcriptional activity of genes involved in TE silencing mechanisms during *X. laevis* early development in the S subgenome. **A**. Expression values of genes encoding KRAB-ZFPs. **B**. Expression values of genes encoding proteins involved in the formation of the heterochromatin and NuRD complex. **C**. Expression values of genes encoding proteins of Argonaute subfamily. The broken line along the Y axis indicates the range of values from 300 to 700 TPM

Our analyses showed that TEs were not completely repressed during *X. laevis* early development, therefore, we investigated the transcriptional activity of genes encoding proteins involved in TE controlling systems. The Krüppel-associated box domain zinc finger proteins (KRAB-ZFPs) are involved in the silencing of TEs binding the TE sequence with the C-terminal array of zinc finger motifs and the TRIM28 protein with the N-terminal KRAB domain. TRIM28 retrieves the heterochromatinization proteins (CBX1, CBX3, and CBX5), the DNA methyltransferases (DNMT1 and DNMT3A), the histone methyltransferase SETDB1, and the nucleosome remodelling deacetylase (NuRD) complex. This latter contains CHD3, CHD4, HDAC1, HDAC2, MBD2, MBD3, MTA1, MTA2, GATAD2A, GATAD2B, RBBP4, and RBBP7. For each protein, we identified two related gene copies located in the L and S subgenome, respectively. Regarding *KRAB-ZNFs* we found 14 genes localized in the L subgenome. With the exception of *KRAB-ZNF 665 L* whose expression was almost undetectable, *KRAB-ZNF 248*,* 234*,* 684*,* 84 L*,* 71*,* 347*,* 783*,* 208*,* 282*, and *250* showed ubiquitous expression across analysed developmental stages, while the remaining *KRAB-ZNF* genes presented a patchy expression (Fig. 7A). Among genes of heterochromatinization, the L subgenome forms of *cbx3*, *cbx5*, *dnmt1*, and *setdb1* were transcriptionally active from zygote to early tailbud, while *cbx1* was transcribed mainly in the last three stages and the expression levels of *dnmt3a* were slightly appreciable (Fig. 7B). *Trim28* as well as all genes encoding proteins of the NuRD complex showed expression across the considered developmental stages with the exception of *chd3* and *mbd2*. Regarding the four *argonaute* genes, the expression of the forms present in the L subgenome were detected during *Xenopus* development (Fig. 7C). In the S subgenome nine *KRAB-ZNF* genes were retrieved and six of them were homoeolog to those located in the L subgenome (*KRAB-ZNF 81*,* 250*,* 577*,* 282*,* 684*, and *665 L*). *KRAB-ZNF 250* and *684* were expressed in all considered stages as well as while *KRAB-ZNF 81*,* 282*,* 41*, and *177* even if at lower levels; *KRAB-ZNF 577* was mainly expressed in the first three analysed stages and *KRAB-ZNF 665 L* and *782* were scarcely transcribed from neurula to early tailbud (Fig. 8A). The homoeolog genes involved in heterochromatinization located in the S subgenome showed the same expression patterns of those of the L subgenome (Fig. 8B). In addition, transcriptional levels were detected for *trim28* and genes of the NuRD complex including *chd3* and *mbd2* that were partially expressed across *Xenopus* development (Fig. 8B). The expression profiles of *argonaute* genes were similar to those of genes located in the L subgenome (Fig. 8C).

## Discussion

Polyploidy seems to be pervasive in all lineages of higher plants while it is less common in animals. In vertebrates, two rounds of whole genome duplication occurred in the ancestor and specific events were reported at the base of teleosts and in several lineages of this clade, as in salmonids and cyprinids [27]. Polyploidy can be distinguished in autopolyploidy if the set of chromosomes doubles within a single species and allopolyploidy if genomes increase the chromosome set after interspecific hybridization of two or more distinct diploid species followed by genome doubling [28]. In the latter case, the beneficial traits of the parent species are mixed contributing to increase the genetic diversity and adaptability [29]. The allotetraploid genome of the African clawed frog *X. laevis* originated through interspecific hybridization of diploid progenitors. The two derived subgenomes have been maintained as separate and distinguishable sets of chromosomes, known as S and L. They differ in the dimensions as well as in gene expression levels and epigenetic status. In particular, the S subgenome showed higher mutation and deletion rates and less selection against the loss of genetic elements leading to shorter chromosomes and lower gene expression [30]. This condition was observed also in plants [31–35] and yeasts [36] in which the dominant subgenome retains more genes (biased fractionation) and shows higher overall gene expression (transcriptomic dominance) compared to the submissive subgenome [37]. The genome dominance has been correlated with the differential suppression of genes near TEs: subgenome with lower density of methylated TEs localized near genes expresses higher RNA levels [37, 38]. Recently, in synthesized Brassica allotetraploids it has been reported no negative relationship between transposon/methylation level and subgenome dominance suggesting that the expression bias might be due to other reasons as species-level differences in the transcription efficiency [39]. It is doubtful that polyploidization events are associated with the reactivation of TEs in hybrids that could have generated strong perturbations [40]. After allopolyploidization, TE transcription can be induced or their copy number can increase compared to their related diploid species [41]. Suda and colleagues [42] have speculated that the activation of DNA transposons and repression of their corresponding piRNAs at the time of hybridization of the two *Xenopus* genomes might have been responsible for the asymmetric evolution between the L and S subgenomes in *X. laevis*.

Although TEs were long referred as “selfish genomic elements”, it is now appreciated their role as drivers of genome evolution, genome organization, and gene regulation. In *X. laevis*, 87% of subgenome-specific enhancers overlap and are associated with annotated repeats, indicating that TEs are active players of regulatory network in this species [30].

Among vertebrates, amphibians show the widest range of genome size (0.95 to 120.60 pg/N) [43, 44] with a variable propensity to accumulate repetitive sequences. *X. laevis* has a genome of 3.5 pg/N and TEs accounted more than half (55%). Its mobilome was mostly dominated by DNA transposons, followed by LINE and LTR retrotransposons; in contrast, SINE retrotransposons represented a restricted portion. Considering the subgenome-specific TEs, LINE retrotransposons were the most abundant. In general, the Kimura landscape showed young TE copies and recent bursts of amplification. This might be explained considering that the genome of *X. laevis* was the result of one of the most recent vertebrate genome duplication events [1]. Moreover, the low presence of remnant TE copies could also due to a remarkable DNA elimination rate in *X. laevis*. Indeed, Elurbe and colleagues [30] have reported an ongoing process of erosion acting on genes, regulatory elements, and genomic sequences, with a stronger bias in the S subgenome than the L subgenome. This led to discrepancies also in the TE number that resulted in fewer copies in the S subgenome compared to the L subgenome.

It is now recognized that TEs can serve as regulatory elements during early embryogenesis and their expression is associated with key developmental progressions [45]. During embryogenesis, TEs escape the transcriptional repression acted by the host since they find a permissive chromatin environment [46]. Indeed, lower epigenetic restriction provides a window of opportunity for transcriptional reactivation of TEs. Mutations induced by TEs might contribute to adaptive evolution influencing the function or expression of genes. TEs are recognized as facilitators of genome evolution creating genetic novelties or regulatory elements. It is noteworthy that novel TE insertions arising during early developmental stages can be responsible for genetic variation inherited from new generations through germline cells. Here, we explored the impact of TE transcriptional contribution in six developmental stages of *X. laevis*. The quantification of TE expression presents several challenges due to the TE features as their repetitive nature, sequence polymorphisms, and diversity in terms of transcript typology [47]. The pipeline here employed minimizes the first two aspects but presents limitations regarding the incapacity to distinguish read-through TE transcription, an issue that will benefit of long-read approaches in the future. Our findings showed activity in zygote that can be related to maternally deposited RNAs, while the heightened value detectable in blastula were ascribable to post zygotic genome activation. The TE transcriptional contribution decreased in gastrula and progressively increased to early tailbud. Overall, the expression pattern referred to the entire set of transcribed TEs was constant across the six developmental stages and in line with their abundance in the genome. Indeed, DNA transposons, LINE and LTR retrotransposons were strongly represented in the transcriptional output suggesting a key role during embryogenesis in *X. laevis*. This result was in line with the expression analyses of the developmental stages pairwise comparisons in which specific elements attributable to DNA transposons, LINE, and LTR retrotransposons showed a differentially significant expression, indicating a stage-specific role for these elements.

The two subgenomes shared a substantial fraction of TE copies that were not distinguishable from each other either by position or expression. Therefore, we focused on those elements that were specific of each subgenome. Our analyses identified 368 TEs unique to the L subgenome and 109 TEs specific to the S subgenome. The presence of TEs specific of each subgenomes has been reported also by Session et al. [1] supporting the idea that the L and S chromosomes originated from two distinct diploid progenitor species. Therefore, the specific TEs were already present in the genomes of progenitors and can be considered as markers of allotetraploidy. Although the TE type composition was similar between the two subgenomes, the transcription pattern across the analysed developmental stages was different. This suggested that differences between the two subgenomes existed also at TE transcriptional level and this could have a diverse impact on the host gene regulatory networks. In particular, it was interesting the activity of LTR retrotransposons specific in the L subgenome after the post-zygotic genome activation, from blastula to early tailbud and LINE retrotransposons in the S subgenome. Subgenome-specific DETEs attributable to these TE typologies were highlighted also in the expression analyses of the developmental stages pairwise comparisons. It has been reported that retrotransposons are considered valuable substrates for evolving new regulatory elements that can be coopted by the host genome to regulate the transcription state of the genome [48, 49]. Moreover, the subgenome-specific retrotransposons transcribed during early *X. laevis* development were young copies. In human and mouse, specific young LTR retrotransposons have been coopted for enhancer function playing a key role in species-specific differences in gene expression observed during embryogenesis [49–52]. It has also been speculated that during embryogenesis, when there is a widespread epigenomic de-repression, TEs direct the transcription of themselves in germ cells to ensure their propagation through vertical transmission.

The expression of LTR retrotransposons and in particular of ERV is controlled by Krüppel Associated box Zinc-finger proteins (KRAB-ZFPs) that bind TEs with their C- terminal zinc fingers and TRIM28 (KAP1) with their N-terminal KRAB domain. TRIM28 facilitates the deposition of repressive heterochromatin at the targeted ERVs recruiting the chromobox protein homologs, the DNA methyltransferases, the histone methyltransferase, and the nucleosome remodeling and deacetylase complex. In primates, this system has been reported to be active in early embryonic development and in adult tissues governing several biological and physiological events [53–59]. Moreover, the activity of genes encoding these proteins in relation to TEs has been investigated in several vertebrates [26, 60–62] and in amphibians most of published works has regarded salamanders in relation to TE load and huge genome size of these organisms [60, 61].

In *X. laevis* we identified 14 genes localized in the L subgenome and nine genes in the S subgenome showing the evolutionarily conserved KRAB domain. Some of *KRAB-ZNF* genes showed ubiquitous expression across analysed developmental stages, others presented a stage-specific expression, and others did not show any transcription. The ubiquitous expression of some *KRAB-ZNF* genes might indicate a key role during embryonic development. Born and colleagues [63] have proposed the ability of KRAB domain to bind TRIM28 enrolling chromatin modifiers to confer transcriptional repression in *X. laevis*. This was also in line with the conservation of this function even in distant lineages, such as coelacanths, proposed by Helleboid and colleagues [64]. Therefore, also in *Xenopus* the KRAB/TRIM28 complex might be employed to recruit proteins involved in heterochromatin formation and those belonging to NuRD complex. Our analyses revealed that, with the exception of *dnmt3a*, genes encoding proteins involved in heterochromatinization were expressed by the homoeolog copies present in the L and S subgenome. Moreover, also genes encoding proteins of NuRD complex showed activity in both subgenomes. The expression of *chd3* and *mbd2* in some stages suggested that they were not constituents of *Xenopus* NuRD complex as observed by Christov and colleagues [65]. Differently, we detected expression levels for *hdac2* and *mta1* indicating that these two proteins might be part of the *Xenopus* NuRD complex. Overall, these findings suggested that an epigenetic remodelling was active and allowed speculating that this system could serve for controlling young copies of LTR retrotransposons in *X. laevis*. Changes in the expression of retrotransposons in *X. laevis* during embryogenesis were related to H3K9me3 deposition [8], a repression mark that in humans is due to KRAB system [59, 66].

Contrarily to humans in which KRAB-ZFPs represent the largest families of transcriptional regulators, in relation to the high amount of retrotransposons, the limited number of *KRAB-ZNFs* found in *X. laevis* could be related to the restricted copy numbers of retrotransposons in its genome.

The patchy expression pattern of some genes encoding KRAB-ZFPs might be explained considering that these proteins can have stage-specific roles or were involved in other functions than transcriptional regulation in *Xenopus* [64, 67].

Our analyses revealed transcriptional activity of genes encoding AGO proteins suggesting that not only systems based on chromatin and histone modifications but also those using small RNAs might be involved in TE regulation during *Xenopus* development. In this regard, Wilczynska and colleagues [68] have reported also the expression of two PIWI genes in early embryo of *Xenopus*. The coexistence of multiple TE repressive mechanisms that potentially act in transposon-selective and developmental stage-specific manner has been suggested also in *X. tropicalis* embryos [69]. Overall, the presence of distinctive mechanisms in the two subgenomes indicated that this condition was already present in the progenitors of *X. laevis* and the asymmetric evolution seems not to have involved the TE controlling systems, probably because of the presence of subgenome-specific TEs. Although, TE type composition between the two subgenomes was similar, we detected differences in the transcription pattern across the analysed developmental stages suggesting a diverse impact on the host gene regulatory networks. Moreover, the investigated TE controlling mechanisms equally cooperate to mediate transposable activity in *X. laevis* in the two subgenomes.

## Electronic supplementary material

Below is the link to the electronic supplementary material.

Supplementary Material 1

Supplementary Material 2

Supplementary Material 3

Supplementary Material 4

## Data Availability

No datasets were generated or analysed during the current study.

## Abbreviations

cbx1: chromobox homolog 1
cbx3: chromobox homolog 3
cbx5: chromobox homolog 5
chd3: chromodomain helicase DNA binding protein 3
chd4: chromodomain helicase DNA binding protein 4
DETEs: Differentially Expressed Transposable Elements
dnmt1: DNA (cytosine-5-)-methyltransferase 1
dnmt3a: DNA (cytosine-5-)-methyltransferase 3 a
gatad2a: GATA zinc finger domain containing 2a
gatad2b: GATA zinc finger domain containing 2b
hdac1: histone deacetylase 1
hdac2: histone deacetylase 2
KRAB: Krüppel box-associated
KRAB-ZFPs: Krüppel box-associated zinc finger proteins
KRAB-ZNF: Krüppel box-associated zinc finger
LINE: Long Interspersed Nuclear Element
LTR: Long Terminal Repeat
mbd2: methyl-CpG binding domain protein 2
mbd3: methyl-CpG binding domain protein 3
mta1: metastasis associated 1
mta2: metastasis associated 1 family, member 2
NCBI: National Center for Biotechnology Information
NuRD: Nucleosome Remodeling Deacetylase Complex
rbbp4: retinoblastoma binding protein 4
rbbp7: retinoblastoma binding protein 7
setdb1: SET domain bifurcated histone lysine methyltransferase 1
SINE: Short Interspersed Nuclear Element
SRA: Sequence Read Archive
TE: Transposable element
TRIM28: Tripartite Motif protein 28
ZFPs: Zinc Finger Proteins
ZNF: Zinc Finger
