# Supplementary material for: Transposable element dynamics in Xenopus laevis embryogenesis: a tale of two coexisting subgenomes
Source: Mob DNA. 2025 Apr 9;16:17. doi: 10.1186/s13100-025-00350-3 (PMC11980090; doi:10.1186/s13100-025-00350-3)
Supplement: Supplementary file 1 — Supplementary Material 1 [file 13100_2025_350_MOESM1_ESM.pdf]

Supplementary material

Transposable element dynamics in *Xenopus laevis* embryogenesis: a tale of two coexisting subgenomes

Edith Tittarelli[1,2], Elisa Carotti[*1], Federica Carducci[1], Marco Barucca[1], Adriana Canapa[1], Maria Assunta Biscotti[1].

1 Dipartimento di Scienze della Vita e dell'Ambiente, Università Politecnica delle Marche, Via Brecce Bianche, 60131, Ancona (Italy).

2 Scuola Universitaria Superiore Pavia – IUSS, Piazza della Vittoria n.15, 27100, Pavia (Italy).

[*]Corresponding author e-mail: e.carotti@univpm.it

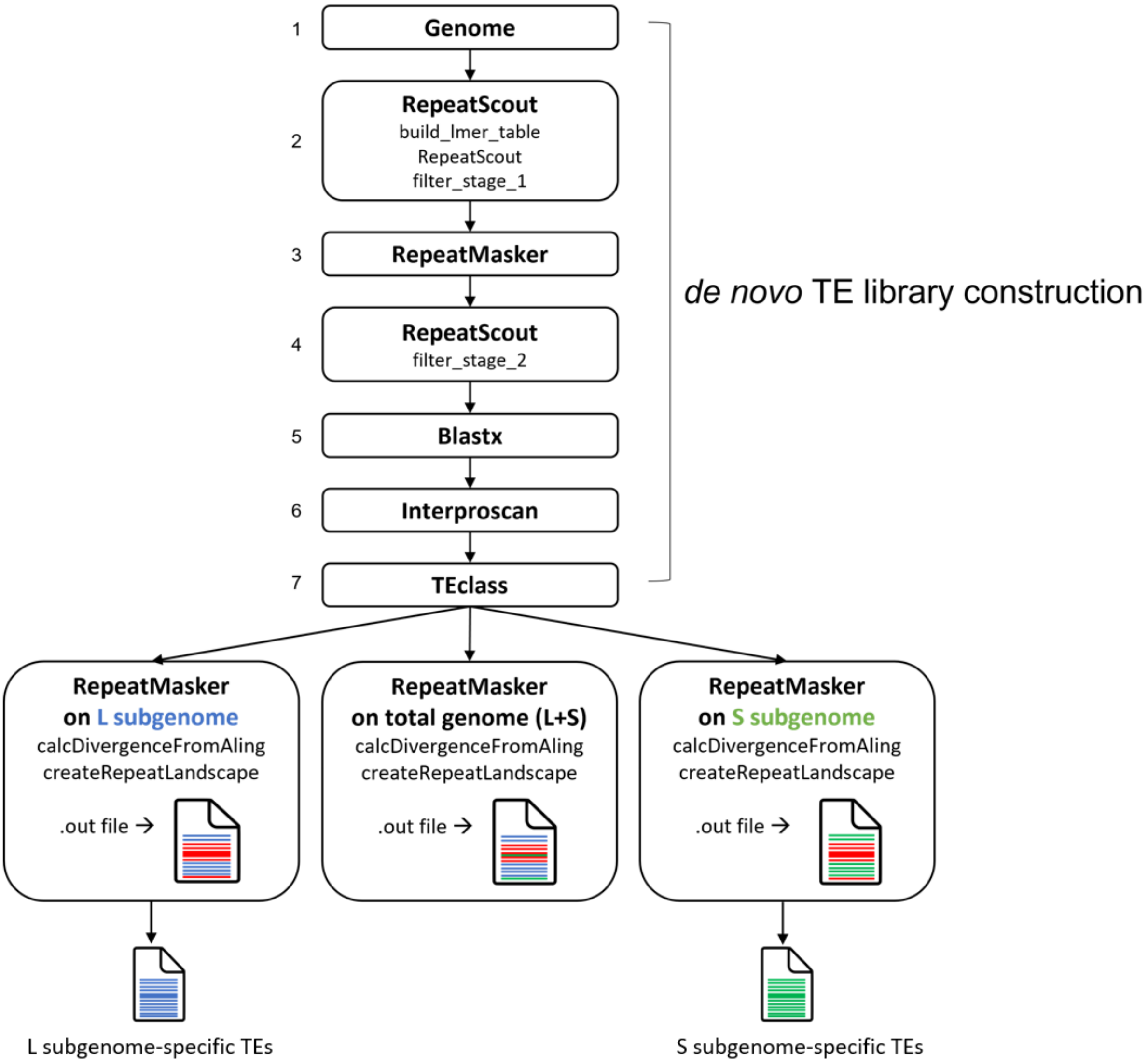

Supplementary Figure 1. Details of bioinformatic workflow for TE annotation at genome and subgenome level. Steps from 1 to 7 are referred to the de novo TE library construction. In steps 2 and 4 the scripts and tools used are specified. In the last RepeatMasker step are specified the tools to calculate the estimation of Kimura distance and create the TE landscape html plot. In the same step, the document icon represents the output file generated by RepeatMasker which contain a list of

classified TE sequences. This includes common TE elements (red lines) shared between L and S subgenomes, and subgenome-specific TEs (blue lines for L subgenome and green lines for S subgenome). The document icon indicates the output file in which only the subgenome-specific TEs L and S are present, blue and green respectively.

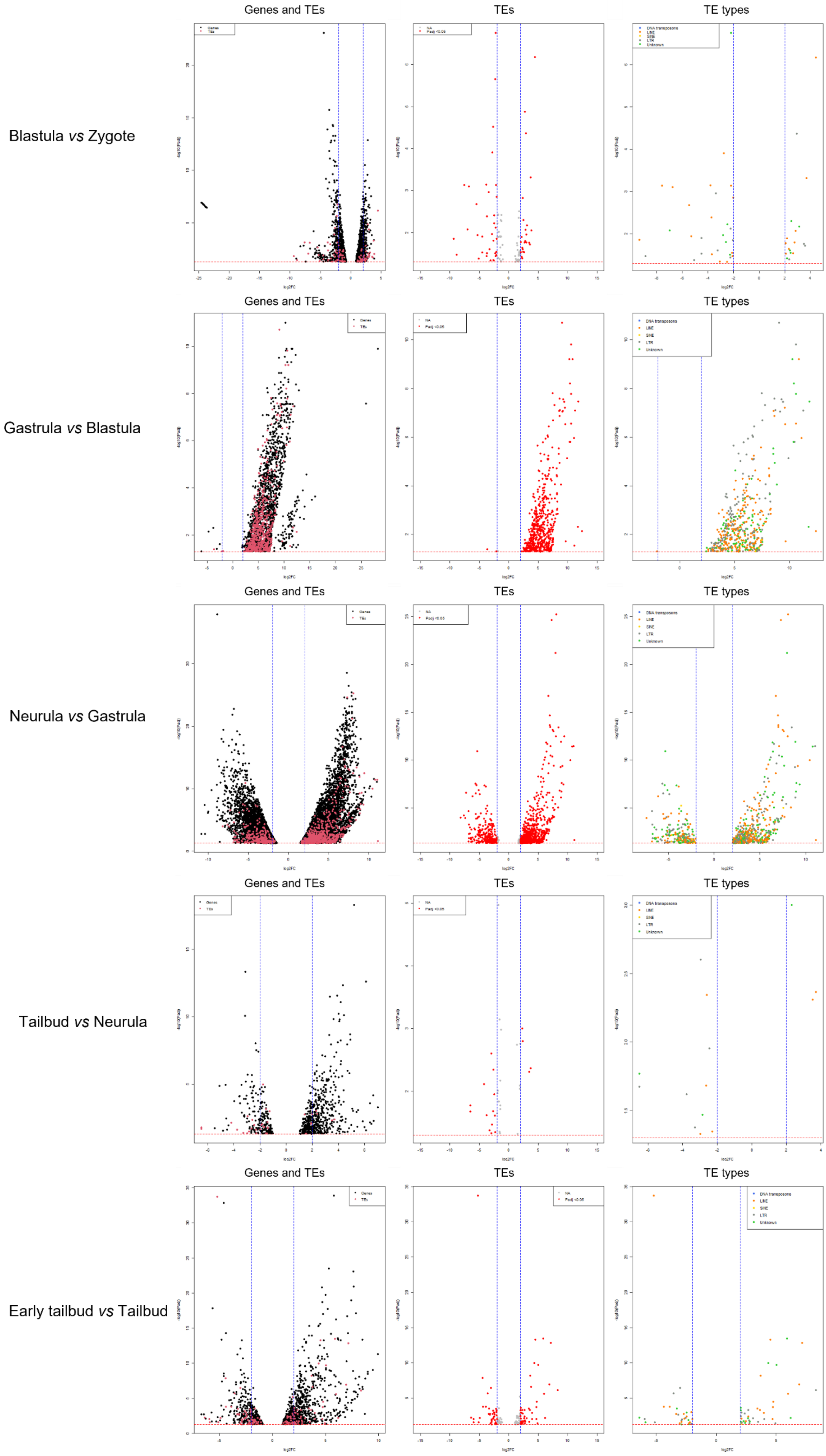
Genes and TEs
TEs
TE types
Blastula vs Zygote
Gastrula vs Blastula
Neurula vs Gastrula
Tailbud vs Neurula
Early tailbud vs Tailbud

Supplementary Figure 2. Volcano plot of differentially expressed TEs (DETEs) during developmental stages of *X. laevis*. Each row is referred to the comparison between two developmental stages (from up to down: Blastula *vs* Zygote, Gastrula *vs* Blastula, Neurula *vs* Gastrula, Tailbud *vs* Gastrula, and Early tailbud *vs* Tailbud). The first column is referred to genes and TEs, the second column is referred uniquely to TEs (significant DETEs in red and not significant DETEs in light grey), and the third column is referred to TE types (DNA transposons in blue, LINE retroelements in orange, LTR retroelements in grey, SINE retroelements in yellow, Unknown, referred to TEs that are not classified as the previous typologies, in green). The blue dashed lines indicate the significant thresholds for Log2 Fold Change > |2|, while the red dashed the statistically significant threshold (-Log10 (p-adj) = 0.05).

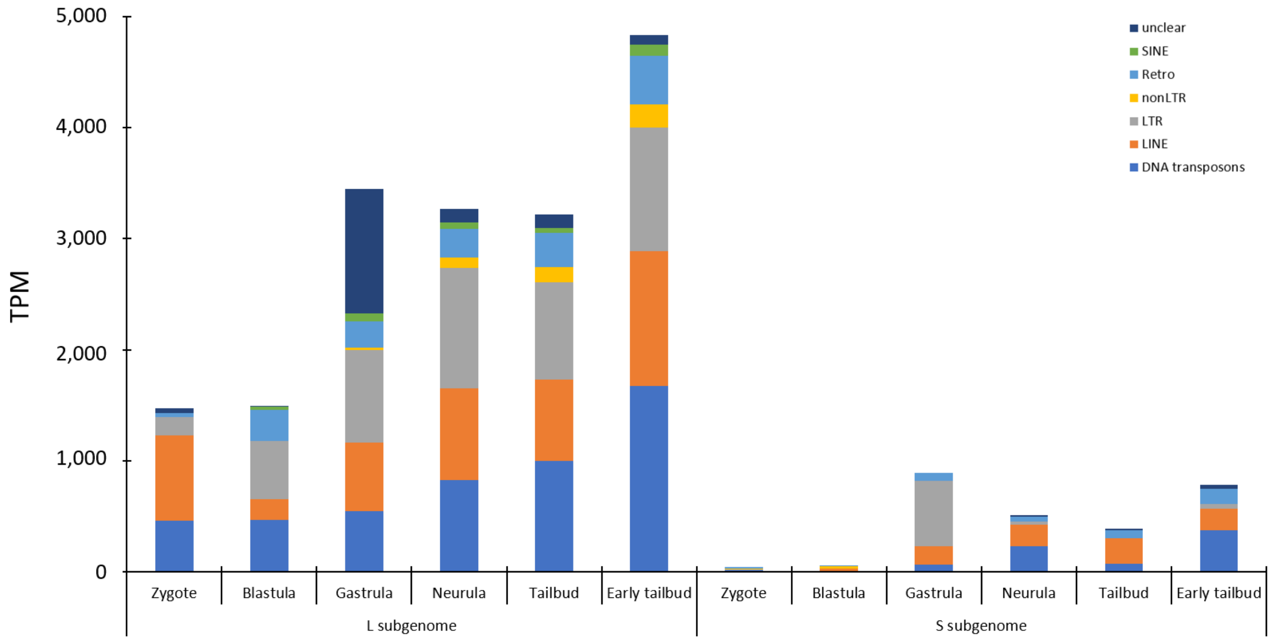

Supplementary Figure 3. Cumulative transcriptional activity of subgenome-specific TEs. On the left, cumulative transcriptional levels of L subgenome-specific TEs were reported; On the right, cumulative transcriptional levels of S subgenome-specific TEs were reported. "Unclear" means TEs that are not classified as DNA transposons, LINE, SINE, and LTR retroelements, "non-LTR" retroelements are referred to retroelements that are not classified as LINE or SINE retroelements, and "Retro" is referred to retroelements that are not classified as LINE, SINE, LTR or non-LTR retroelements.

				SRA Access code
**Zygote**	**stage 1**	SRX19887604	RNA-seq of Xenopus Laevis: embryo stage 1 rep 1	SRR24086762
		SRX19887605	RNA-seq of Xenopus Laevis: embryo stage 1 rep 2	SRR24086761
		SRX19887606	RNA-seq of Xenopus Laevis: embryo stage 1 rep 3	SRR24086760
**Blastula**	**stage 8**	SRX19891541	RNA-seq of Xenopus Laevis: embryo stage 8 rep 1	SRR24090892
		SRX19891532	RNA-seq of Xenopus Laevis: embryo stage 8 rep 2	SRR24090901
		SRX19891533	RNA-seq of Xenopus Laevis: embryo stage 8 rep 3	SRR24090900
**Gastrula**	**stage 10**	SRX19890515	RNA-seq of Xenopus Laevis: embryo stage 10 rep 1	SRR24089878
		SRX19890516	RNA-seq of Xenopus Laevis: embryo stage 10 rep 2	SRR24089877
		SRX19890517	RNA-seq of Xenopus Laevis: embryo stage 10 rep 3	SRR24089876
**Neurula**	**stage 18**	SRX19893082	RNA-seq of Xenopus Laevis: embryo stage 18 rep 1	SRR24092470
		SRX19893083	RNA-seq of Xenopus Laevis: embryo stage 18 rep 2	SRR24092469
		SRX19893084	RNA-seq of Xenopus Laevis: embryo stage 18 rep 3	SRR24092468
**Tailbud**	**stage 22**	SRX19893085	RNA-seq of Xenopus Laevis: embryo stage 22 rep 1	SRR24092467
		SRX19893086	RNA-seq of Xenopus Laevis: embryo stage 22 rep 2	SRR24092466
		SRX19893087	RNA-seq of Xenopus Laevis: embryo stage 22 rep 3	SRR24092465
**Early tailbud**	**stage 28**	SRX19893088	RNA-seq of Xenopus Laevis: embryo stage 28 rep 1	SRR24092464
		SRX19893089	RNA-seq of Xenopus Laevis: embryo stage 28 rep 2	SRR24092463
		SRX19893090	RNA-seq of Xenopus Laevis: embryo stage 28 rep 3	SRR24092462

Supplementary Table 1. Accession numbers of RNA-seq data analyzed and related *Xenopus laevis* developmental stages.

Supplementary Table 2. Gene and TE expression tables for the five comparisons between *Xenopus laevis* developmental stages. Each sheet reports data regarding the single comparison performed with TEtranscripts.

Supplementary File 1. TE annotation of *Xenopus laevis* genome.

Supplementary File 2. TE consensus library of *Xenopus laevis* genome.
